# The Potential of Double Blinding with Two Placebo Acupuncture Needles: A Randomized Controlled Pilot-Trial

**DOI:** 10.3390/medicines2010011

**Published:** 2014-12-30

**Authors:** Miho Takayama, Hiroyoshi Yajima, Akiko Kawase, Ikuo Homma, Masahiko Izumizaki, Nobuari Takakura

**Affiliations:** 1 Department of Acupuncture and Moxibustion, Faculty of Health Sciences, Tokyo Ariake University of Medical and Health Sciences, 2-9-1 Ariake, Koto-ku, Tokyo 135-0063, Japan; E-Mails: takayama@tau.ac.jp (M.T.); yajima@tau.ac.jp (H.Y.); 2 Department of Physiology, Showa University School of Medicine, 1-5-8 Hatanodai, Shinagawa-ku, Tokyo 142-8555, Japan; E-Mails: kawase@hanada.ac.jp (A.K.); hommai@tau.ac.jp (I.H.); masahiko@med.showa-u.ac.jp (M.I.); 3 Japan School of Acupuncture, Moxibustion and Physiotherapy, 20-1 Sakuragaokacho, Shibuya-ku, Tokyo 150-0031, Japan; 4 The Foundation for Oriental Medicine Research, 28-9 Sakuragaokacho, Shibuya-ku, Tokyo 150-0031, Japan; 5 Department of Nursing, Faculty of Nursing, Tokyo Ariake University of Medical and Health Sciences, 2-9-1 Ariake, Koto-ku, Tokyo 135-0063, Japan

**Keywords:** acupuncture, double blind, placebo, randomized controlled study, complementary and alternative medicine

## Abstract

Background: Whether acupuncture treatment employing multiple penetrating, skin-touch placebo, or no-touch placebo needles designed for double blinding actually do blind practitioners and patients has not been investigated. We aimed to investigate this question. Subjects: 120 patients with functional neck/shoulder stiffness but in otherwise healthy condition were randomly assigned to a treatment using four penetrating, four skin-touch placebo, or four no-touch placebo needles. Each of six acupuncturists applied four needles to four acupoints in the neck/shoulder of 20 patients. Acupuncturists and patients were asked to guess the treatment mode and their confidence in their guesses on 100 mm visual analog scales. Results: The kappa coefficients between practitioner guesses and treatment type and between patient guesses and treatment type were 0.15 and 0.44, respectively. The median score of practitioner confidence was 46.8, and no significant difference in confidence between correct and incorrect guesses was revealed for any treatment. The median score of patient confidence for correct guesses was 77.6. The kappa coefficient between practitioner and patient guesses was 0.06. Conclusions: The practitioners were blinded to the nature of treatment using the same multiple needles, but patient blinding was insufficient. Further improvement is necessary to achieve satisfactory patient blinding with these acupuncture needles.

## 1. Introduction

The efficacy of complementary and alternative medicine (CAM) must be determined via randomized, controlled trials (RCT) to gain general acceptance as evidence-based medicine. RCTs are generally considered the gold standard for distinguishing specific effects of intervention from non-specific effects [1,2]. Furthermore, where technically possible, double blinding is essential when investigating any therapies. Acupuncture is one of the most popular CAM therapies, and single blind (patient blinding) acupuncture placebo/sham needles have been developed to allow acupuncture studies to more closely achieve the most rigorous methodological standards [3,4]. These needles are the best possible blinding devices for clinical acupuncture trials [5,6]; however, they are not designed to blind acupuncturists to the administration of a real or placebo/sham needle. In acupuncture research, double blinding had been considered almost impossible to achieve because blinding an acupuncturist seemed impossible due to the nature of the procedure [7,8,9]. This unblinding of practitioners in single blind studies produces an undeniable potential bias [10,11,12,13,14,15]. We designed skin-touch placebo needles for double (practitioner-patient) blinding with matched penetrating needles and evaluated the effectiveness of these needles in an attempt to alleviate the methodological difficulty of blinding practitioners [16,17,18,19].

In previous validation studies for double blinding using the penetrating and skin-touch placebo needles, at least half of the guesses made by experienced acupuncturists after each needle application did not coincide with the nature of needles [16,17,18]. Furthermore, we designed no-touch placebo needles, where the needle tip does not reach the skin, to settle the controversy of whether skin-touch placebo needles are true placebos [9,20,21,22,23,24]. The no-touch placebo needles effectively blinded practitioners [22]. When acupuncture-experienced subjects were treated with the skin-touch placebo and penetrating needles, they incorrectly guessed half of the skin-touch placebo needles as penetrating [18]. Furthermore, more than 30% of penetrating needles were guessed as skin-touch placebos by the subjects [17,18]. However, full patient blinding became difficult to achieve when the no-touch placebo needle was used [23,24], although most subjects were not certain of the accuracy of their guesses for no-touch placebo needles and penetrating needles [23,24]. We believe that these needles are the best safeguard against bias in acupuncture studies.

In our previous validation studies, practitioners and subjects were asked to guess the type of needle used after each needle application. Therefore, the question remains as to whether a treatment using multiple needles and repeated applications can blind practitioners and patients in clinical trials. In the present study, practitioners and subjects guessed the type of needle used during a treatment consisting of four needles of the same type after all needle applications. The aim of this study was to assess the potential to blind practitioners and patients to treatments of multiple penetrating, skin-touch placebo, or no-touch placebo needles designed for double blinding in randomized controlled trials.

## 2. Methods

### 2.1. Study Design

We conducted a randomized placebo-controlled trial with three treatment modes: treatment with penetrating needles, treatment with skin-touch placebo needles, and treatment with no-touch placebo needles (see supplementary Table S1).

The study protocol was fully explained to each practitioner and patient using a written explanation of the study protocol, including the details of the needles, and participants provided written consent before treatment. The ethics committee of the Showa University School of Medicine approved the study. Trial registration: ISRCTN34405634 [25].

### 2.2. Setting and Participants

The study was conducted at the Japan School of Acupuncture, Moxibustion and Physiotherapy, Tokyo, Japan.

We employed six (three males, three females) licensed and experienced acupuncturists (experience of acupuncture practice, mean ± standard deviation: 12.5 ± 11.8 years). We recruited 120 patients with functional neck/shoulder stiffness (mean ± standard deviation: 29.7 ± 9.3 years of age, 62 males and 58 females). Patients were otherwise in a healthy condition and were acupuncture students. We assumed that blinding acupuncture students who know needle sensations well is more difficult than blinding ordinary people. We asked patients about their condition and the results of their annual medical examinations. Patients with any diseases that may be the cause of their neck/shoulder stiffness or pain, such as high blood pressure or cervicobrachial syndrome, were excluded from this study.

### 2.3. Interventions

#### 2.3.1. The Needles

We used three types of needles for double blinding: 1) penetrating needles that penetrate the skin; 2) skin-touch placebo needles, the tip of which presses against the skin but cannot penetrate it; and 3) no-touch placebo needles, the tip of which does not reach the skin. The appearances of these three types of needles are indistinguishable [22,23,24]. The diameter of the needles was 0.16 mm. The insertion depth of the penetrating needle was 5 mm. These details have been described previously [16,17,18,19,22,23,24]. A pedestal for the needles was not used in this study.

#### 2.3.2. The Outer Guide Tube for Tapping-In Method

**Figure 1:**
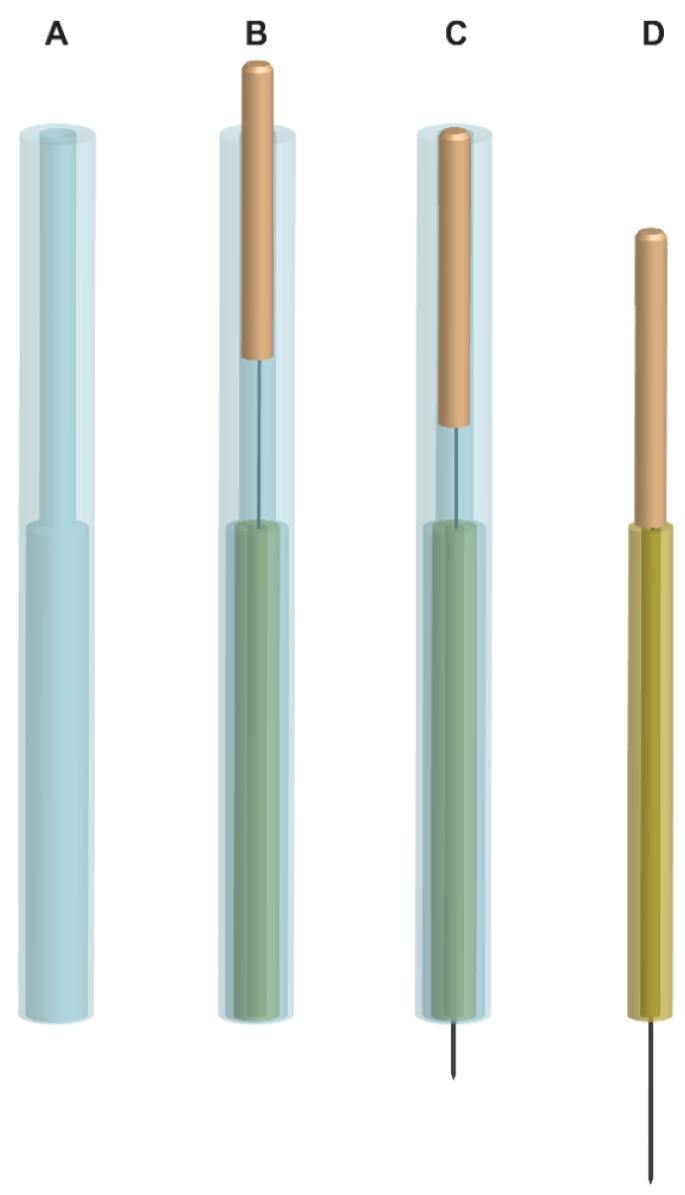
Penetrating needle for double blinding with outer guide tube. (**A**) Outer guide tube; (**B**) Before tapping-in method. The upper part of the needle handle protrudes from the outer guide tube by 4 mm. The inner diameter of the upper part of the outer guide tube from the top of the inner tube is slightly larger than the diameter of the needle handle, and the inner diameter of the rest of the lower part of the outer guide tube is slightly larger than the outer diameter of the inner opaque tube, which stabilizes the needle handle in the outer guide tube during the tapping-in method application; (**C**) After tapping-in method. The protruding part (4 mm) of the needle handle is inserted into the outer guide tube using the tapping-in method to penetrate the skin. (For the skin-touch and the no-touch placebo needle, needle tips penetrate the lower stuff in the inner tube at this stage.); (**D**) Needle insertion after removal of the outer guide tube. After the tapping-in method, the outer guide tube is removed, and the needle is inserted using the alternating twirling technique. The inner diameter of the inner tube is smaller than the diameter of the needle handle so that the inner tube works as a stopper. (The blunt tip of the skin-touch needle slightly protrudes from the inner tube to touch the skin but not for the no-touch placebo needle at this stage).

We designed an outer guide tube for this study (Figure 1A) to improve the usability of the needles to employ the tapping-in method, which is a Japanese art of skin penetration [24]. The acupuncturists practiced the tapping-in method using the outer guide tube before the acupuncture treatments to familiarize themselves with the technique.

#### 2.3.3. Randomization

**Figure 2:**
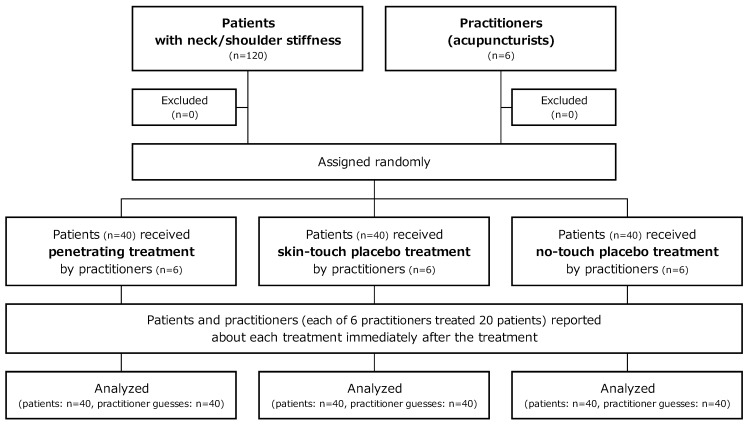
Flowchart.

We prepared 40 sets of four penetrating needles (penetrating treatment), 40 sets of four skin-touch placebo needles (skin-touch placebo treatment), and 40 sets of four no-touch placebo needles (no-touch placebo treatment). We prepared 120 bags for sterilization that were numbered from 1 to 120. A study controller randomly assigned the 120 numbered bags to the three treatment types, *i.e.*, penetrating, skin-touch placebo, and no-touch placebo, using a table of random numbers that was generated by the RAND function (Microsoft Office Excel 2007). Four penetrating needles, four skin-touch placebo needles, or four no-touch placebo needles were put in a numbered bag corresponding to the assigned treatment type and sealed by the study controller. The sealed bags were sterilized with gaseous ethylene oxide. Each of the six acupuncturists treated 20 patients consecutively. The first to the last patient were assigned to the 120 numbered bags in order. The 120 patients were randomly assigned to penetrating, skin-touch placebo, or no-touch placebo treatment as shown in Figure 2. Thus the six acupuncturists were randomly assigned to the three types of treatments among the 20 patients.

### 2.4. Protocol

The entire protocol was described (see below) to the practitioners and patients before treatment. We told practitioners and patients that penetrating, skin-touch placebo, or no-touch placebo needles would be used for individual treatments and provided a written explanation and images of the needles. We did not tell them that equal numbers of penetrating, skin-touch, and no-touch treatments would be used in this study. We also told the patients of the possibility that they may suffer adverse effects with the treatment, such as pain, uncomfortable sensation, slight cerebral anemia, external bleeding, and internal bleeding.

**Figure 3:**
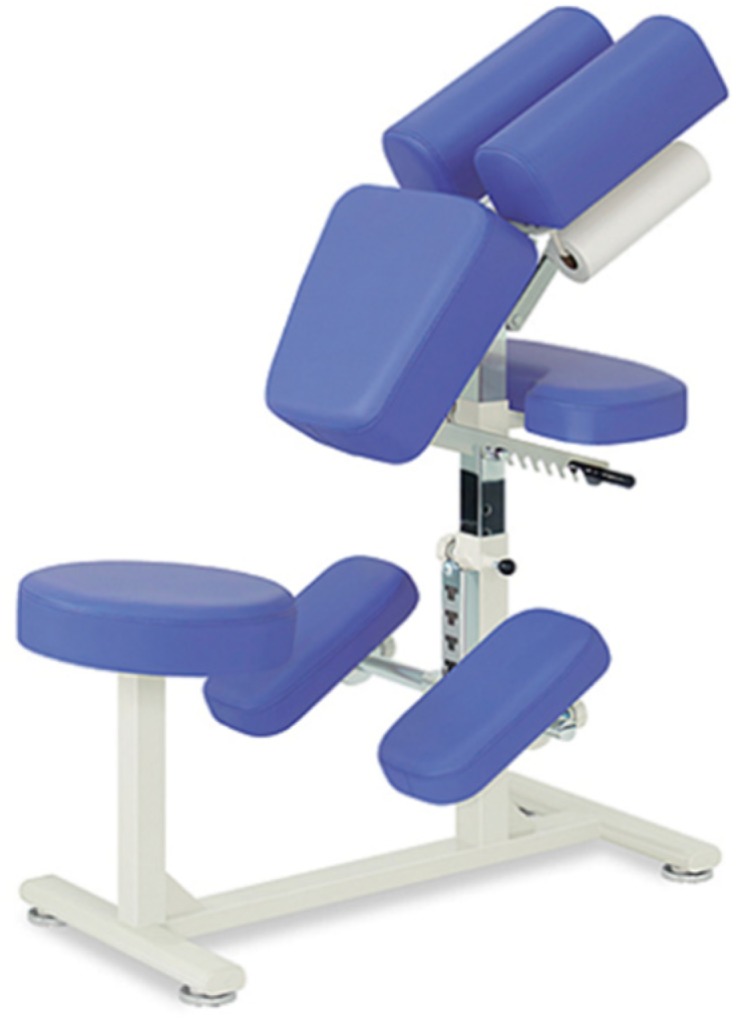
The chair used for acupuncture treatment.

**Figure 4:**
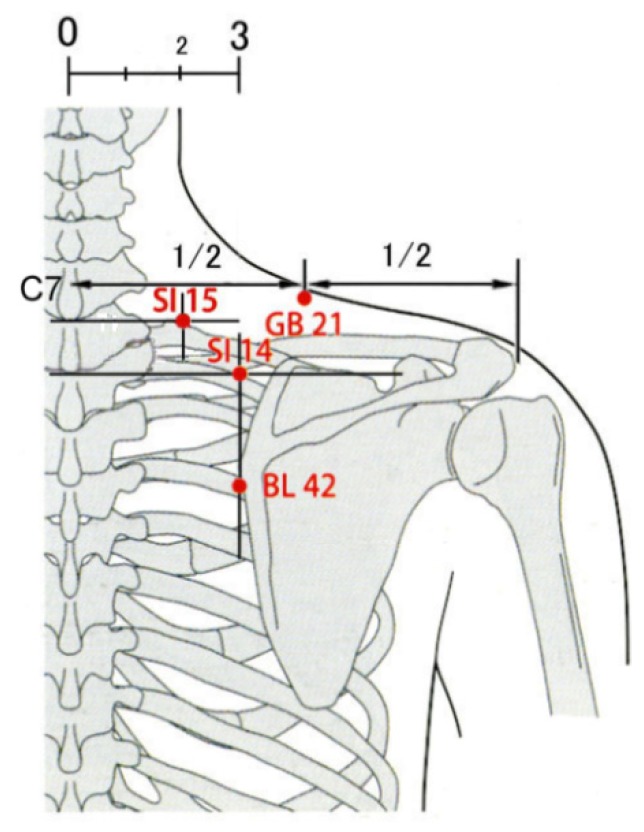
Acupoints of SI14, SI15, GB21, and BL42.

Patients were guided into a treatment booth by an assistant (assistant-1), and they sat on a chair (Figure 3) in which they could put their face and chest facing downward on the pads of the chair, which helped them to relax. An acupuncture-licensed assistant (assistant-2) located acupoints of the small intestine (SI)14, SI15, gallbladder (GB)21 and bladder (BL)42 [26] on the right neck/shoulder (Figure 4) of patients to apply needles using a light touch. Using alcohol-soaked cotton, assistant-2 sterilized the skin on which the acupoints were located. Assistant-1 handed four needles to a practitioner one at a time. The practitioner placed a needle assembly with the outer guide tube (Figure 1B) on an acupoint and tapped the upper end of the needle handle protruding from the top of the outer guide tube to penetrate the skin or the lower stuff (Figure 1C) using the index finger pulp (tapping-in method). The practitioner removed the outer guide tube after completion of the tapping-in method to insert the needle further (Figure 1D) by alternately rotating the needle clockwise and counterclockwise (alternating twirling technique). Each needle was inserted until the needle tip reached the target depth, and the needle was returned immediately to its initial position (simple insertion) using the alternating twirling technique [16,17,18,22,23,24]. The needle assembly was removed from the skin and placed into an opaque envelope by the practitioner. Assistant-1 sealed the envelope. Practitioners and patients answered a self-completed questionnaire after treatment with four needles that asked about the nature of the treatment (see the Section 2.5 Outcome Measurements). The practitioners, assistant-1, and assistant-2 maintained the same approach with each application, engaged in the same conversations with every patient, and they remained blinded to the type of needle throughout the experiment. The practitioners and assistant-1 monitored the patients for the presence of adverse events, such as pneumothorax, bleeding, hematoma, dizziness, tiredness, and needle pain.

### 2.5. Outcome Measurements

The practitioners were asked to guess the type of treatment applied, and they also reported their confidence in their guesses on a 100 mm visual analog scale (VAS) ranging from 0 (no confidence) to 100 (full confidence) [22,23,24]. The practitioners reported on the clues as to their guesses, such as patient expression, behaviors, external bleeding, internal bleeding, no bleeding, feeling of needle insertion, feeling of needle removal, and other factors on a multiple-answers questionnaire [22,23,24].

The patients were also asked to guess which type of treatment they received after treatment completion, and they also reported their confidence in their guesses of the treatment on the 100 mm VAS [23,24].

Patients rated the pain intensity associated with the treatment on a 100 mm VAS ranging from 0 (no pain) to 100 (most intense pain imaginable). The pain intensity rating was used in the analysis to examine its association with type of treatment, patient guesses and patients’ confidence in their guesses [23,24].

### 2.6. Statistical Analysis

We used the kappa coefficient to measure the level of agreement between the six practitioners in regard to their guesses (excluding the “unidentified”) about what type of needle was being applied and actual treatments. Individual practitioner guesses (excluding the “unidentified”) and the patient guesses were also measured in this way. The chi-squared test of independence was used to compare the number of each treatment type assigned to each practitioner. It was also used to compare the number of penetrating treatment, skin-touch placebo treatment, and no-touch placebo treatment guesses for each acupuncturist, and also among the six acupuncturists. The chi-squared test of goodness-of-fit was used to determine whether the numbers of correct and incorrect guesses fitted an expected probability. The Kruskal-Wallis test was used to detect significant differences among the three treatment groups. The Mann-Whitney’s U test was used to identify pair-wise group differences in the confidence levels of practitioners and patients guesses and in the pain intensity associated with treatment. We used the kappa coefficient to measure inter-rater agreement between practitioner guesses (excluding the “unidentified”) and patient guesses. The Spearman’s rank correlation coefficient measured the relationship between two variables. The chi-squared test of independence compared the results according to gender and grades of school as baseline characteristics. The Kruskal-Wallis test compared the three treatments according to patient age. All statistical analyses were performed using IBM SPSS Statistics 20 (SPSS Japan Inc., an IBM company, Tokyo, Japan) [22,23,24].

## 3. Results

The study began in January 2009, and was completed in February 2009. The flow of the subjects during the study is shown in Figure 2. A total of 120 randomized participants and six acupuncturists completed the study. Baseline characteristics did not differ significantly in the three treatment groups (Table 1).

**Table 1:** Baseline characteristics of participants.

Patient characteristics			40 penetrating treatments	40 skin-touch placebo treatments	40 no-touch placebo treatments
120 patients	Age in years (mean ± standard deviation)		31.2 ± 10.3	28.9 ± 9.7	29.0 ± 7.7
	Sex (number)	Male	19	21	20
		Female	21	19	20
	Grades at school (number)	1 year	17	21	20
		2 year	11	10	11
		3 year	12	9	9

### 3.1. Practitioner Blinding

For each practitioner, the years of experience as acupuncturist, the number of allocated treatment types, and their guesses are shown in Table 2. There was no significant difference in the rate of allocation to the three types of treatments among the six acupuncturists (chi-square value = 2.40, *p* = 0.99). The kappa coefficients between individual practitioner guesses (excluding the “unidentified”) and the treatments assigned to them were 0.05 to 0.26, respectively, indicating “poor” strength of agreement [27]. Furthermore, for every individual practitioner, the chi-squared test of independence revealed no significant difference in the numbers of penetrating, skin-touch placebo, and no-touch placebo guesses or “unidentified” guesses among penetrating, skin-touch placebo, and no-touch placebo treatment. On the other hand, there was a significant difference in the numbers of penetrating, skin-touch placebo, and no-touch placebo guesses among the six practitioners (chi-square value = 57.0, *p* < 0.01), which indicated that the practitioner guesses were done on the basis of their own intuition and each had their individual tendencies.

Practitioner guesses and confidence in those guesses on a 100 mm VAS are shown in Table 3. The kappa coefficient between the guesses of the six practitioners (excluding the “unidentified”) and the actual treatment modes was 0.15 [95% confidence interval (CI): 0.03 to 0.28] which indicates “poor” strength of agreement [27]. Two penetrating and one no-touch placebo treatment were correctly guessed with full confidence. One skin-touch placebo treatment was guessed with full confidence, but it was not coincident with the practitioner’s guess. The median score (mean ± standard deviation) of practitioners’ confidence was 46.8 (43.8 ± 28.8). There was no significant difference in the practitioners’ confidence between correct and incorrect guesses for the respective treatments (penetrating treatment: *p* = 0.24, skin-touch placebo treatment: *p* = 0.40, no-touch placebo treatment: *p* = 0.15), which indicated that the confidence levels were unrelated to whether or not the practitioner guess was correct or incorrect.

**Table 2:** Practitioner guesses associated with the three treatment modes.

Practitioner (Experience as acupuncturist)	Type of treatment	Number of practitioner guesses				Total
		Guessed as penetrating treatment	Guessed as skin-touch treatment	Guessed as no-touch treatment	Unidentified	
**1 **(5 years) *Κ* = 0.05 (−0.30, 0.40) ^† ^χ^2^ = 9.5 (*p* = 0.15) ^¶^	Penetrating treatments	1	4	0	2	7
	Skin-touch placebo treatments	0	1	4	1	6
	No-touch placebo treatments	0	1	3	3	7
	Total	1	6	7	6	20
**2 **(7 years) *Κ* = 0.15 (−0.21, 0.52) ^† ^χ^2^ = 10.2 (*p* = 0.12) ^¶^	Penetrating treatments	1	3	1	2	7
	Skin-touch placebo treatments	3	1	1	2	7
	No-touch placebo treatments	0	0	4	2	6
	Total	4	4	6	6	20
**3 **(35 years) *Κ* = 0.26 (0.01, 0.52) ^† ^χ^2^ = 9.9 (*p* = 0.13) ^¶^	Penetrating treatments	2	0	3	1	6
	Skin-touch placebo treatments	0	1	6	0	7
	No-touch placebo treatments	0	0	7	0	7
	Total	2	1	16	1	20
**4 **(3 years) *Κ* = 0.10 (−0.22, 0.43) ^† ^χ^2^ = 4.4 (*p* = 0.35) ^¶^	Penetrating treatments	3	2	2	0	7
	Skin-touch placebo treatments	0	2	4	0	6
	No-touch placebo treatments	1	3	3	0	7
	Total	4	7	9	0	20
**5 **(10 years) *Κ* = 0.12 (−0.14, 0.37) ^† ^χ^2^ = 6.9 (*p* = 0.14) ^¶^	Penetrating treatments	2	2	3	0	7
	Skin-touch placebo treatments	0	0	5	0	5
	No-touch placebo treatments	1	0	7	0	8
	Total	3	2	15	0	20
**6 **(15 years) *Κ* = 0.18 (−0.05, 0.41) ^† ^χ^2^ = 3.6 (*p* = 0.46) ^¶^	Penetrating treatments	5	0	1	0	6
	Skin-touch placebo treatments	5	0	3	1	9
	No-touch placebo treatments	2	0	3	0	5
	Total	12	0	7	1	20

**^†^** Kappa coefficient (*Κ*) (95% confidence interval) between individual practitioner guesses excluding “unidentified” responses; ^¶^ Pearson’s chi-square value (*p* value) for comparison of guess numbers among the three treatments for each practitioner.

Most treatments were guessed on the basis of the feeling of the needle insertion or/and removal, and more than half of the guesses using these clues did not coincide with the actual treatment (Table 4). The respective numbers of correct *versus* incorrect guesses using the feeling of the needle insertion, by the feeling of needle removal, and both were 34:45 (*p* = 0.07), 18:25 (*p* = 0.24), and 42:57 (*p* = 0.06) respectively; these fit an expected 1:2 ratio.

**Table 3:** Number and confidence level of practitioner guesses associated with the three treatment modes. (Note: Bold numbers indicate correct guesses).

Treatment	Practitioner guesses (number (median, mean ± SD of confidence level on 100 mm VAS))				Total
	Guessed as penetrating treatment	Guessed as skin-touch treatment	Guessed as no-touch treatment	Unidentified	
**Penetrating treatments**	**14** (36.1, 45.8 ± 30.7)	11 (47.0, 50.2 ± 15.7)	10 (57.8, 57.1 ± 24.6)	5 (0)	40 (42.3, 44.1 ± 28.7)
**Skin-touch placebo treatments**	8 (13.0, 20.9 ± 16.3)	**5 **(39.6, 37.2 ± 17.6)	23 (53.7, 55.4 ± 25.1)	4 (0)	40 (44.7, 40.7 ± 28.6)
**No-touch placebo treatments**	4 (28.4, 35.1 ± 33.4)	4 (47.5, 46.6 ± 16.8)	**27** (56.5, 56.9 ± 24.5)	5 (0)	40 (50.0, 46.6 ± 29.6)
**Total**	26 (30.4, 36.5 ± 28.7)	20 (42.9, 46.3 ± 16.4)	60 (55.9, 56.4 ± 24.4)	14 (0)	120 (46.8, 43.8 ± 28.8)

**Table 4:** The relationship between clues and practitioner guesses. (Note: Some of the clues noted by patients overlap those listed above.)

Clues for guessing type of acupuncture treatment	Practitioner guesses		Total
	Correct guesses	Incorrect guesses	
Facial expression	1	1	2
Verbal expression	1	2	3
Bleeding and internal bleeding	6	0	6
No bleeding	0	2	2
Feeling of needle insertion	34	45	79
Feeling of needle removal	18	25	43

**Table 5:** Number and confidence level of patient guesses for the three treatment modes. (Note: Bold numbers indicate correct guesses).

Treatment	Patient guesses (number (median, mean ± SD of confidence level on 100 mm VAS))			
	Guessed as penetrating treatment	Guessed as skin-touch treatment	Guessed as no-touch treatment	Total
**Penetrating treatments**	**27** (84.8, 81.3 ± 18.4)	13 (70.6, 62.7 ± 19.2)	0 (—)	40 (75.9, 75.2 ± 20.4)
**Skin-touch placebo treatments**	17 (73.9, 69.7 ± 29.2)	**22** (50.0, 60.6 ± 16.5)	1 (58.8)	40 (58.0, 64.4 ± 22.7)
**No-touch placebo treatments**	5 (50.0, 53.4 ± 25.8)	9 (74.1, 70.5 ± 21.0)	**26** (78.1, 78.5 ± 18.1)	40 (77.6, 73.5 ± 21.0)
**Total**	49 (78.4, 74.4 ± 24.6)	44 (63.8, 63.2 ± 18.2)	27 (78.0, 77.7 ± 18.2)	120 (75.3, 71.1 ± 21.7)

### 3.2. Patient Blinding

Patient guesses and their confidence levels are shown in Table 5. The kappa coefficient between the patient guesses and actual treatment modes was 0.44 (95% CI: 0.31 to 0.57) which indicates “moderate” strength of agreement [27]. The median patient confidence in their correct guesses was 77.6. Nine penetrating and three no-touch placebo treatments were correctly guessed with full confidence. Five skin-touch placebo treatments and one no-touch placebo treatment were incorrectly guessed with full confidence. The patient confidence in their correct guesses was significantly larger than for incorrect guesses in penetrating treatment (*p* = 0.01) and no-touch placebo treatment (*p* = 0.04).

For the intensity of pain associated with the three treatment modes, there were significant differences between the penetrating, skin-touch placebo, and no-touch placebo treatments (*p* < 0.01) (Table 6). However, there was no significant difference in patient confidence according to treatment mode (*p* = 0.08) (Table 5). Furthermore, there was no significant correlation between pain intensity and patient confidence in either the penetrating (*r* = 0.18, *p* = 0.26), skin-touch placebo (*r* = 0.05, *p* = 0.77), or no-touch placebo (*r* = −0.18, *p* = 0.27) modes.

### 3.3. Relationship between Practitioner and Patient Blinding

The kappa coefficient was 0.06 (95% CI: −0.05 to 0.17) between practitioner and patient guesses (excluding the “unidentified”) and this indicated “poor” strength of agreement [27] (Table 7). Of the 120 treatments, 11 penetrating, two skin-touch placebo, and 18 no-touch placebo treatments were correctly guessed by both practitioners and patients. There were no treatments for which both practitioner and patient were completely confident. Patient confidence level was significantly larger than practitioner confidence level (*p* < 0.01). There was no significant correlation between practitioner and patient confidence levels in 31 treatment applications correctly guessed by both (*r* = −0.08, *p* = 0.68).

**Table 6:** Pain intensity with treatments associated with patient guesses.

Treatment	Patient guesses (median, mean ± SD pain intensity on 100 mm VAS)			
	Guessed as penetrating treatment	Guessed as skin-touch treatment	Guessed as no-touch treatment	Total
**Penetrating treatments**	15.7, 21.9 ± 22.8	13.2, 17.5 ± 16.5	-	14.5, 20.4 ± 20.8
**Skin-touch placebo treatments**	4.9, 8.7 ± 12.4	4.3, 6.4 ± 11.7	1.6	4.3, 7.3 ± 11.8
**No-touch placebo treatments**	2.0, 2.9 ± 2.6	1.4, 2.7 ± 4.0	0.0, 0.8 ± 1.9	0.0, 1.5 ± 2.7
**Total**	6.8, 15.4 ± 19.7	4.3, 8.9 ± 13.4	0.0, 0.8 ± 1.9	3.3, 9.7 ± 15.9

**Table 7:** The relationship between practitioner and patient guesses.

Practitioner \ Patient		Number of patient guesses			Total
		Guessed as penetrating treatment	Guessed as skin-touch treatment	Guessed as no-touch treatment	
**Number of practitioner guesses**	**Guessed as penetrating treatment**	12	11	3	26
	**Guessed as skin-touch treatment**	11	6	3	20
	**Guessed as no-touch treatment**	20	22	18	60
	**Unidentified**	6	5	3	14
**Total**		49	44	27	120

### 3.4. Safety

There were no serious adverse events reported during the study period. Six patients experienced slight transient bleeding after needle removal.

## 4. Discussion

We assessed the potential to blind practitioners and patients to acupuncture treatment modes using multiple penetrating, skin-touch placebo, and no-touch placebo needles. The kappa value for practitioner treatment mode guesses showed poor agreement. The confidence level for correct patient guesses was larger than for correct practitioner guesses, and the kappa value for patient treatment mode guesses indicated a moderate level of agreement. These results suggest the practitioners were blinded to the treatment mode using these needles, but patient blinding was insufficient. Considering that our patients were acupuncture students, treatment mode was guessed without complete confidence and their confidence in their incorrect guesses was also high, so these acupuncture needles do have potential for double blinding in clinical trials. However, further studies are needed to improve the effectiveness of double blinding with acupuncture needles used in this study to achieve satisfactory patient blinding.

For practitioner blinding, the kappa value showed a poor level of accuracy, and practitioners with more years of acupuncture experience did not make more correct guesses; the low certainty of the practitioner guesses support the conclusion that practitioners were blinded to treatment mode using the multiple needles. Correct guessing was not predictable from the level of practitioner confidence because of the low certainty of their guesses of being correct, and there was no significant difference in their confidence when making correct and incorrect guesses. The majority of the needles were guessed from “feeling of needle insertion or/and removal,” and the respective numbers of correct and incorrect guesses using these feelings through needle manipulation fit an ideal ratio, which means it was inherently difficult for acupuncturists to identify the treatment mode using the same multiple needles. Furthermore, the number of penetrating, skin-touch placebo, and no-touch placebo guesses or unidentified guesses among penetrating, skin-touch placebo and no-touch placebo treatment for each practitioner was not statistically different; but was different among the six practitioners. This indicated that practitioners actually guessed by their own individual perception, which may be different from other practitioners. These results suggest that the needles used in this study for double blinding are a useful tool for practitioner blinding in clinical trials.

For patients, the kappa value showed a moderate level of agreement, and their confidence in their correct guesses was greater than that of the practitioners. This suggests on the face of it that patient blinding was not sufficient. Given the relatively large number of treatment applications that were correctly guessed with high confidence by patients, we must conclude that it was difficult to fully blind patients to the treatment mode using the same needles. Patient blinding is inherently difficult in acupuncture treatment, because penetrating and skin-touch needles induce patient sensations but no-touch needles do not; for example, this is quite different to pill administration *versus* placebo administration where the sensation is the same. Therefore, we believe that the patients in this study had a relatively high confidence in their guesses regardless of whether they were accurate or not. In our validation study series, we chose acupuncture students as subjects because they were experienced in receiving acupuncture and knew needle sensations well, which means they should be much more difficult to blind than the general public. Considering the difficultly in achieving full double blinding in pharmacological randomized trials [28] and the added difficulty (skin sensations) acupuncture poses for double blinding, it may be the reasonable results that the majority of the patients were not completely confident about their guesses with the close kappa coefficient to the fair level of accuracy. It must be emphasized that patients who incorrectly guessed treatment mode did appear to have high confidence when they were informed that they would receive either penetrating, skin-touch placebo, or no-touch placebo acupuncture. Furthermore, the kappa coefficient between practitioner and patient guesses was very close to 0, which means patient confidence in correct guesses did not affect practitioner guesses and vice versa. In fact, there were no treatments guessed with full confidence for both the practitioner and the patient. Given these findings, acupuncture needles for double blinding may have a limitation for patient blinding to some extent, and therefore, complete patient blinding using these needles cannot be guaranteed.

Given that the kappa value for patient blinding in this study was at a moderate level of accuracy, we must be aware of impartial guesses and treatment outcomes that may show bias. Unavoidable needle sensations or the absence of sensations felt by patients gives the patient a relatively strong positive or negative guide as to the treatment given. We believe analysis of the treatment outcome according to the patient guess is essential to understanding the effectiveness of acupuncture. If practitioner blinding can be achieved in an acupuncture study, subgroup analysis would be meaningful to investigate the healing power of the patient mind. For example, we could undertake a patient subgroup analysis using the nine groups in a 3 × 3 factorial assortment, e.g., penetrating, skin-touch placebo, or no-touch placebo treatment paired with either “penetrating,” “skin-touch,” or “no-touch” in patient guess. This may reveal the specific effects of acupuncture, placebo effects on patients, and the efficacy of acupuncture in routine clinical care. Practitioner blinding is crucial to enable us to understand the healing power of the patient mind entangled with needle insertion. From this perspective, in blinded acupuncture studies it is informative to ask patients what treatment they thought they received and how certain they felt that their guess was correct.

In the Japanese style of acupuncture, the simple insertion technique, whereby the needle is removed immediately after needle insertion to the desired depth, is commonly used in real treatment as well as the needle retention technique, whereby inserted needles reach the desired depth and are maintained in place for a desired time [29]. We did not use pedestals to set up and erect the needle at an acupoint in this study, because we employed only the simple insertion technique to keep needle application in the same way as in real treatment of neck/shoulder stiffness as much as possible [29]. The patient blinding effects may have been affected with the use of pedestals; however, there has been no study in which practitioners and subjects guessed various treatment modes using the same multiple needles with a pedestal after completion of all needle applications as in the present study. Therefore, findings from the present study cannot be compared with previous study findings [22,23,24] and consistency cannot be established. Further validation research using these acupuncture needles with pedestals and employing the needle retention technique are needed to investigate whether the skin-touch stimulation with the pedestal has an influence on patient blinding.

We employed the tapping-in method to penetrate the skin using an outer guide tube, and then the needle is inserted further using the alternating twirling technique according to the Japanese style of acupuncture. We designed an outer guide tube to improve the usability of the needles for double blinding to employ the tapping-in method for smooth, fast, and easy skin penetration [24]. In fact, when using the tapping-in method even without a guide tube, blinding is apparently improved and the frequency of painless skin penetration with the penetrating needles is increased compared with the twirling-in method [23,24]. The difficulty in employing the tapping-in method with needles for blinding trials [24] was overcome by using the outer guide tube, and therefore, treatments employing these needles have become much more similar to ordinary acupuncture treatment.

This study had the following limitations. We did not administer continuous treatments, and therefore, we must validate the blinding effect of the needles for practitioners and patients using multiple treatments. Insertion with the penetrating needles was exclusively 5 mm to the neck/shoulder. The needles were removed immediately after needle insertion to the specific depth, and therefore, in future studies, the needles should be validated when they remain and are manipulated in the body. The number of acupuncturists was relatively small; therefore, the conclusion on practitioner blinding should be carefully interpreted. The subjects were exclusively acupuncture students who had a moderate knowledge of acupuncture, and they were not asked to report the clues used in making their guesses. Therefore, the blinding efficacy on patients should be tested with acupuncture-naïve subjects and with consideration of the various clues the patients use to identify the needles. Further research is necessary to address these limitations and to improve these needles into more effective double blind acupuncture devices by considering practitioner and patient clues used for guesses.

## 5. Conclusions

The practitioners were blinded to the nature of treatments using the same multiple needles, but patient blinding was insufficient. Further studies are needed so that improvements can be made to achieve satisfactory patient blinding and to increase the effectiveness of double blinding with these acupuncture needles.

## Supplementary Materials

Supplementary File 1
